# Adolescent Soccer Overuse Injuries: A Review of Epidemiology, Risk Factors, and Management

**DOI:** 10.3390/ijerph22091388

**Published:** 2025-09-05

**Authors:** Adam Ayoub, Maxwell Ranger, Melody Longmire, Karen Bovid

**Affiliations:** Homer Stryker MD School of Medicine, Western Michigan University, Kalamazoo, MI 49008, USA; adam.ayoub@wmed.edu (A.A.); maxwell.ranger@wmed.edu (M.R.);

**Keywords:** youth soccer, overuse injuries, growth-related injury risk

## Abstract

Introduction: Overuse injuries are a growing concern among adolescent soccer players, with the repetitive nature of the sport placing significant physical demands on young athletes. These injuries can have long-term implications for physical development, performance, and overall well-being. This narrative synthesis aimed to evaluate the existing literature on the epidemiology, risk factors, and management strategies for overuse injuries in adolescent soccer players. Methods: A comprehensive literature search was conducted using PubMed and Embase. A total of 123 articles were identified, 27 of which met the inclusion criteria after screening. Studies focusing on overuse injuries in adolescent soccer players aged 10–18 years were included, while those addressing acute injuries, non-soccer populations, or adult athletes were excluded. Relevant quantitative and qualitative data were extracted and evaluated. Due to heterogeneity in study designs and outcomes, findings were narratively synthesized rather than meta-analyzed. Results: The period around peak height velocity (PHV: 11.5 years in girls, 13.5 years in boys) was consistently identified as a high-risk window, with seven studies demonstrating a significantly increased incidence of overuse injuries. Additional risk factors included leg length asymmetry, truncal weakness, early sport specialization, high ratios of organized-to-free play, and increased body size. Injury burden was greatest for hamstring and groin injuries, often leading to prolonged time lost from play. Preventive interventions such as plyometric training, trunk stabilization, and structured load monitoring demonstrated reductions in injury incidence in several prospective studies, though protocols varied widely. Conclusion: This narrative synthesis highlights PHV as the most consistent risk factor for overuse injuries in adolescent soccer players, alongside modifiable contributors such as training load, sport specialization, and free play balance. Evidence supports neuromuscular training and structured monitoring as promising preventive strategies, but there remains a lack of standardized, evidence-based protocols. Future research should focus on optimizing and validating interventions, integrating growth and load monitoring, and leveraging emerging approaches such as machine learning-based risk prediction.

## 1. Introduction

Adolescent soccer players are at an increased risk for musculoskeletal injuries due to the high physical demands of training and competition coupled with decreased ability of tissues including the growth plates to withstand load during periods of rapid growth. Overuse injuries, which result from repetitive mechanical stress without sufficient recovery, are particularly prevalent in this population [1]. Epidemiological studies have reported that between 30 and 50% of youth soccer players experience an overuse injury within a season, with the knee and ankle being the most commonly affected sites [2]. Unlike acute injuries, overuse injuries develop gradually and often go unnoticed until they significantly impact function, making early identification and prevention essential. Given the intensity of elite youth soccer training, understanding the risk factors, common injury locations, and effective management strategies is crucial for optimizing player development and minimizing long-term musculoskeletal complications [3].

Several intrinsic and extrinsic factors contribute to the development of overuse injuries in youth soccer players. Intrinsic factors include neuromuscular imbalances, muscle weakness, and deficits in coordination, which may predispose athletes to inefficient movement patterns and increased joint stress [2]. Extrinsic factors such as excessive training volume, inadequate recovery, and improper workload management further compound injury risk [4]. The knee and ankle are among the most frequently affected sites due to the high demands of running, jumping, kicking, and sudden directional changes involved in soccer. Ligamentous structures, tendons, and growth plates in these areas are particularly vulnerable to repetitive loading, leading to conditions such as patellar tendinopathy, Sever’s disease, and stress fractures [1].

Despite growing recognition of overuse injuries in adolescent soccer players, there remains no standardized approach to prevention and management. Neuromuscular training and plyometric exercises have been shown to improve strength and coordination, potentially reducing injury risk [5]. However, their efficacy in specifically addressing overuse injuries remains uncertain, particularly in growing athletes. Additionally, methods for monitoring training load and detecting early signs of injury vary widely, with no universally accepted guidelines for youth soccer players [6]. Given these gaps in knowledge, this review aims to provide a comprehensive synthesis of the current evidence on the epidemiology, risk factors, and prevention strategies for overuse injuries in adolescent soccer players. By evaluating risk factors and evidence-based management strategies, we seek to inform targeted interventions that enhance long-term athletic development while minimizing injury-related setbacks.

## 2. Methods

This literature review was conducted to evaluate the relationship between overuse injuries, growth-related risk factors, and injury prevention strategies in adolescent soccer players. The study adhered to the Preferred Reporting Items for Systematic Reviews and Meta-Analyses (PRISMA) guidelines to ensure methodological rigor and transparency in study selection, data extraction, and synthesis. The protocol for this review was not registered on PROSPERO or other registries due to its narrative nature; however, the review followed standardized procedures for literature selection and synthesis to maintain transparency.

A comprehensive literature search was performed across PubMed and Embase databases to identify peer-reviewed studies published from 2014 to 2024. This timeframe was chosen to capture the most contemporary evidence, reflecting recent changes in training practices, youth soccer participation trends, and updated injury prevention strategies. Earlier studies were excluded to ensure findings were directly applicable to current clinical and sports settings. The search strategy incorporated a combination of Medical Subject Headings (MeSH) terms and relevant keywords, including “overuse injuries,” “youth soccer,” “growth-related injury risk,” “cumulative trauma disorders,” and “injury prevention.” Boolean operators (AND/OR) were used to refine search results, and additional relevant studies were identified by manually screening reference lists of included articles.

Studies were eligible for inclusion if they investigated overuse injuries in adolescent soccer players aged 10–18 years, explored the influence of growth-related factors such as peak height velocity (PHV), neuromuscular deficits, or training load on injury risk, or assessed prevention strategies, including neuromuscular training, plyometric exercises, or load management. Only studies published in English in peer-reviewed journals were considered. Exclusion criteria included studies focusing primarily on acute traumatic injuries, research conducted on non-soccer athletes or adult populations, and publications such as case reports, editorials, and non-peer-reviewed conference abstracts. A detailed description of the search strategy is provided in Appendix A.

Three independent reviewers screened titles and abstracts for relevance, with full-text articles retrieved for studies meeting the inclusion criteria. Discrepancies between reviewers were resolved through discussion and consensus; when disagreement persisted, a fourth reviewer adjudicated the decision. A standardized data extraction form was used to systematically collect information on study design, participant characteristics, injury definitions, identified risk factors, training interventions, and outcomes.

A formal risk of bias assessment was conducted for included studies. Given the heterogeneity of study designs, we applied the MINORS tool for cross-sectional and interventional studies and a modified Newcastle-Ottawa Scale (NOS) for cohort studies. AMSTAR-2 was utilized for narrative and systematic reviews. Studies were categorized as having low, moderate, or high risk of bias. Although this approach may not capture every nuance of methodological variability, it was chosen for its adaptability across non-randomized study designs.

Given the variability in study designs, participant populations, and outcome measures, a meta-analysis was not performed. Although heterogeneity across studies was the primary limitation to performing a meta-analysis, narrative synthesis was further justified because of the small number of eligible studies, the inconsistent definitions of cumulative trauma disorders, and the diverse outcome reporting in the adolescent soccer population. A meta-analysis under these conditions would risk generating misleading pooled estimates, whereas narrative synthesis allowed us to qualitatively integrate and compare findings while maintaining transparency and validity. Where possible, injury incidence rates, training interventions, and biomechanical risk factors were compared across studies to provide a comprehensive evaluation of current evidence. The results were categorized into thematic domains to facilitate interpretation and provide actionable insights for injury prevention in adolescent soccer players.

## 3. Results

**Figure 1:**
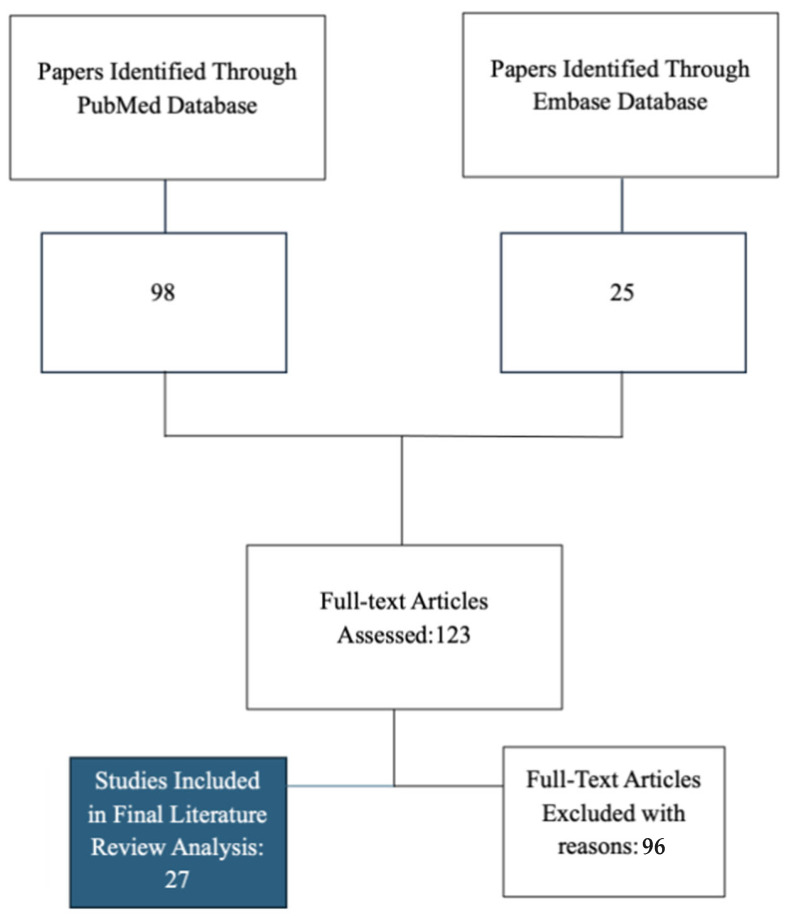
Flow diagram outlining the study selection process. A total of 123 records were identified through database searches (98 from PubMed and 25 from Embase). After screening and application of eligibility criteria, 27 studies were included in the final review. A total of 96 full-text articles were excluded based on the inclusion criteria.

A total of 123 articles were identified through the initial database search. Following the application of inclusion and exclusion criteria, 27 studies were deemed suitable for inclusion in this review (Figure 1) The included studies are summarized in Table 1.

### 3.1. Risk of Bias

Of the 27 included studies, risk of bias varied across study designs. Systematic and narrative reviews were predominantly of moderate risk due to incomplete reporting of search strategies and lack of protocol registration, although some reviews incorporated structured critical appraisals. Prospective cohort studies generally demonstrated low to moderate risk, with limitations primarily related to adjustment for confounding factors, sample size, or outcome assessment methods. Retrospective cohort and survey studies were mostly moderate risk due to potential recall bias, incomplete injury reporting, and variability in exposure measurement. Prospective interventional studies were generally low risk, reflecting rigorous follow-up and objective outcome measurement, although some lacked formal sample size calculations or blinding. Overall, 9 studies (33%) were rated as low risk, 16 studies (59%) as moderate risk, and 2 studies (7%) as high risk of bias. Despite methodological variability, the consistent identification of growth-related risk factors, neuromuscular deficits, and effective injury prevention strategies across low- and moderate-risk studies strengthens confidence in the synthesized findings.

### 3.2. Anatomical Location of Injuries/Epidemiology

Overuse injuries are highly prevalent among adolescent soccer players, accounting for a significant proportion of musculoskeletal complaints in this population [9]. These injuries arise from repetitive mechanical stress on developing musculoskeletal structures, often exacerbated by the high-intensity training and match schedules characteristic of competitive youth soccer. Epidemiological studies have demonstrated that overuse injuries can manifest in various anatomical locations, with the lower extremities being the most affected regions, particularly the knee, ankle, and hip [3,21]. Specifically, one systematic review and meta-analysis of epidemiological data of injuries reported that lower extremity injuries had the highest incidence rate of 6.8 injuries per 1000 h of exposure [21].

Among lower extremity injuries, patellar tendinopathy and Sever’s disease (calcaneal apophysitis) are frequently reported, particularly in adolescent players undergoing rapid growth and increased training loads [27]. Groin injuries, including adductor-related pain, are also common in youth soccer players, with research indicating that age-related variations in muscle forces may contribute to a heightened risk during key developmental stages [7]. Given the multifactorial nature of overuse injuries in youth soccer, targeted injury prevention strategies are essential to mitigate risk and promote long-term musculoskeletal health. Evidence suggests that structured neuromuscular training, appropriate load management, and individualized conditioning programs can play a pivotal role in reducing injury incidence [9].

### 3.3. Risk Factors

Risk factors for overuse injury can be categorized as modifiable or nonmodifiable. Modifiable factors include training type, training volume, sport specialization, and level of fitness. Examples of nonmodifiable factors intrinsic to the athlete are growth, maturation, timing of peak height velocity (PHV), and limb asymmetry.

One of the best documented nonmodifiable risk factors identified in the literature for sustaining overuse injuries is PHV which refers to the period when an individual’s vertical (height) growth is the fastest (excluding the period from age 0–1). This usually occurs during puberty, approximately age 11.5 in girls and age 13.5 in boys [29]. Many studies included in this review used the Maturity Offset Protocol to calculate PHV which is a non-invasive approach that uses an individual’s age, gender, standing height, sitting height and weight to approximate their proximity to PHV without the need for radiographs of growth plates or inspections of genital/pubertal development. This approach requires a high level of accuracy in each measurement to accurately gauge maturity and growth status but is particularly well suited for screening and large sample size studies because of the decreased cost and avoidance of the invasiveness associated with other methods of measuring PHV. Seven studies included in our review found the period around peak height velocity (PHV) to be associated with a significantly higher overuse injury incidence rate [4,10,12,14,16,17,18].

Other analyses that included a related measure of whether individuals could be categorized as “early” or “late” maturing, defined by them reaching PHV before or after average timing showed mixed results. Van Der Sluis et al. found that late maturing players had increased risk of injuries [17], while Johnson et al. found no correlation [4]. This could be due to how the demands of playing and practicing soccer become more intense as youth athletes age, meaning those who experience PHV later are being exposed to more intense stresses during this period of vulnerability to injury. This may also be reflective of the large variation in player sizes, strength, and maturity related to differences in timing of growth in this age group.

Another important measure evaluated in several studies was the players’ injury burden, defined by the amount of time needed to recover from an injury. Injuries to youth athletes can vary widely in the amount of time they cause a player to be sidelined, and prevention of the most debilitating and burdensome injuries should be prioritized. Prior injuries can also increase risk of future injuries, whether due to inadequate time spent recovering, deconditioning from taking time off, or because some injuries never fully heal and are prone to re-injury. Two studies that focused on elite youth athletes found that hamstring injuries and groin muscle sprains were the most burdensome [18,26]. Two studies also found that injury burden increased significantly during players’ PHV [4,18].

Lastly, less established factors like players’ weight may influence risk with one study finding increased weight correlated with overuse injury risk potentially because increased weight increases the amount of stress on the skeleton, making pathological accumulation of microtrauma more likely [18]. However, other studies examining this correlation found insignificant results and even that lower calculated body fat percent was correlated with higher injury risk [10]. Other factors like asymmetries in limb lengths and other skeletal asymmetries have not been studied enough to make claims about how they affect players’ injury risk, primarily because of the expense and radiation exposure needed to measure bone growth accurately. Asymmetries are known to be present in youth athletes especially around PHV and are thought to present sources of increased stress that could contribute to overuse injury development [14].

Modifiable risk factors identified in the literature are related to the amount of soccer played, the intensity of said playing, whether players are single-sport athletes, and the ratio of time spent playing “organized sport” vs. participating in “free play”. While the association between overuse injuries and repetitive mechanical stress suggests that increased playing time correlates with a higher risk of injury, mitigating these injuries extends beyond merely reducing playing time. It is important to consider categorizing soccer activities to distinguish between games (highest intensity and injury risk), practice, and other related activities like stretching, weightlifting or plyometric exercises. Another distinction analyzed in two studies was between organized sport and “free play”, defined as time kids spent playing actively outside of organized sports. Both these studies found a correlation that an increased ratio of time spent in organized sport vs. free play predisposed athletes to higher overuse injury risk [8,15]. Playing other sports at the same time or in the offseason may increase, decrease or not significantly affect risk and this relationship is not yet fully understood and necessitates further exploration [8,15,20].

### 3.4. Management

Given the impact of overuse injuries with pain, need for treatment and recovery, and missed playing time affecting adolescent athletes, there has been interest in the literature in how to best identify, prevent, and manage these conditions and address modifiable risk factors. Some plyometric exercises and measures of strength and flexibility are correlated with decreased risk of overuse injuries [2,5,17], highlighting the need to analyze different aspects of participation in soccer separately to more effectively understand how these injuries develop. Although multiple prevention strategies have been investigated, most studies are descriptive of a single intervention and do not directly compare the effectiveness of different interventions.

One study that screened over 1000 players at a soccer tournament found that while only 26.9% of players with positive exam findings underwent imaging, 75.5% of those imaged were diagnosed with radiographic abnormalities including apophysitis in 90%, 1 case of osteochondritis dissecans of the distal femur and 3 cases of spondylolysis raising concerns that undiagnosed apophysitis exists in many players [28]. This insidious overload may cause increasing symptoms and/or contribute to more burdensome injuries in the future.

Implementing specific exercises or warm-ups before or after practice may help reduce acute and overuse injuries amongst youth soccer players. One systematic review looked at the effectiveness of plyometric training on youth soccer athletes in preventing overuse injuries [5]. Plyometric exercises enhance athletic performance with the utilization of stretch-shortening cycle: technique used to improve power by engaging the stretch reflex and elastic components of tendons and muscles. The reviewed studies suggest that plyometric training completed 2 days per week over 8 to 10 weeks with a 72 h rest interval was the most effective. Programs should start with 50–60-foot contacts per session, increasing to 80–120 max contacts, and a sum of 2–4 plyometric exercises with 2–4 sets for 5–15 repetitions should be included per session. Significant improvements in kicking distance, agility, jumping ability, and sprint times were observed after recommended plyometric training [5]. In the authors’ experience, implementing these plyometric recommendations alongside a basic warmup and stretching routine with a U12 boys soccer team was simple and very manageable requiring approximately 8 min at the beginning of practice twice per week. An assistant coach with training and experience in plyometric training supervised the athletes and taught proper jumping/landing techniques. No additional equipment was required outside of cones that were already being utilized during practice for drills.

Additionally, soccer training sessions negatively impact postural stability [13], but the implementation of specific trunk stabilization warm-up exercises may reduce lower extremity injuries due to the improvement of postural control and balance [22]. Imani et al. observed two male junior soccer teams with one preforming trunk stabilization exercises (INT) before practice and games, and one control team (CON) who followed their usual warm-up. Both teams trained regularly. Overall injury rates were lower in the INT team (2.65 injuries/1000 h) compared to the CON team (4.94 injuries/1000 h). Additionally, acute injuries were lower in the INT team (1.91 injuries/1000 h) compared to the CON team (4.06 injuries/1000 h). Notably, ankle injuries had a significant reduction in the INT team (0.32 injuries/1000 h) compared to the CON team (2.28 injuries/1000 h) [22]. This suggests that trunk stabilization exercises can help prevent injuries in male junior soccer players.

Monitoring biological maturity and training load is crucial in injury prevention, specifically during growth spurts in adolescent athletes [24]. Salter et al. surveyed forty-nine support staff from the female Regional Talent Clubs and male Premier League academies in the UK regarding the current monitoring of the player’s biological maturity and training load. Results indicate inconsistency in the approaches of the methods for estimating biological maturity and tracking training loads. Resource limitations, communication issues, and staff numbers create barriers in effective monitoring practices, specifically with the communication of training load data to medical staff [24]. One example of a more standardized protocol included a machine learning model [12]. A total of 734 players were monitored during one season beginning with preseason anthropometric measurements (height, sitting height, and weight), physical fitness (flexibility, agility, endurance, and strength) used to predict injury risks. Extreme gradient boosting algorithms predicted injury risk with 85% precision, recall, and accuracy, and predicted injury classification (overuse vs. Acute) with 75% precision, recall, and accuracy. Top predictors of injury risk were PHV, greater body height, leg length, lower fat percentage, and average performance on the standing broad jump. The models allow for injury risk management strategies tailored to individual players, potentially improving the efficacy of monitoring injury risk factors for individual players. While physical examinations are crucial in tracking injuries, for elite adolescent female soccer athletes hip physical examinations with a minimum 5-year follow up were not predictive of their subsequent rate of overuse injury [21].

Another study observed a new development strategy called Elite Player Performance Plan (EPPP) that would increase training hours to a total of 8500 h throughout a player’s junior and adult career [25]. The study aimed to identify if the increased soccer exposure has any impact on injury incidence, but despite the increase in training hours, there was a reduction in injury incidence from 3.0 to 2.1 injuries per 1000 h post EPPP. One factor that may have influenced this is that elite soccer academies reported implementing 5 injury prevention sessions per week. Additionally, for age groups 12–15, these players were often given specific individual training programs to reduce their soccer exposure by monitoring maturation and predicting PHV.

Match congestion can also affect hip adductor squeeze strength and groin pain [26]. In this study, a 5 s adductor squeeze strength was recorded during a 7-game international tournament. Adductor strength and time had a negative relationship as 72.7% of the players recorded had a meaningful 15% or more reduction in adductor strength, which can increase the risk of groin pain. Thus, monitoring adductor strength could be included as part of injury prevention during or after a congested tournament.

## 4. Discussion

The most established risk factor for youth athletes developing overuse injuries is proximity to PHV [4,11,12,14,16,17,18]. There are several potential explanations for this correlation; delays and regressions in sensorimotor function associated with growth, maladaptive stress distribution caused by limb asymmetries, the decreased density of new bone growth, and a temporary vulnerability of connective tissues and joints induced by rapid growth [4]. Given the amount of evidence of significantly increased injury risk and burden during this period, sport governance bodies should consider both providing resources to screen for when players are experiencing this vulnerable growth period and managing their time in the highest risk activities like games until they are past PHV. Additionally, closer monitoring of growth-related changes and early detection of biomechanical imbalances and insidious injuries should be considered to help identify at-risk players before injuries become chronic or debilitating. Using a tape measure to obtain standardized measurements would be an inexpensive and accessible way to screen for fast growth or limb length discrepancies

Education about what injuries players are at risk for, how to recognize the early signs, and how to limit risk and protect oneself from injury should be more extensively explored because currently youth athletes receive very little standardized education about their injury risks and there is little research about how effective it could be at reducing risk of injury. Educating players about the anatomy and development of common injuries they might experience or see in peers has the potential of empowering them to be more active in the process of injury prevention. An app or other internet-based system would likely be the most practical way to make education accessible to as many of the people involved as possible in a cost-effective manner. It would also empower individuals to do things like track their own growth and playing time (or their child’s or players’) and receive individualized advice about what limits and protective activities they can use to manage their health. Currently, apps like the Apple health app exist to track exercise, but they are not specific to soccer and lack widespread adoption and backing from major science and sports bodies. Future efforts would ideally include medical authorities and researchers integrating soccer-specific, science-backed guidelines and could appeal for funding and institutional support from sports governance bodies (for example, FIFA). Existing efforts like FIFA 11+ have used such backing to develop a bi-weekly exercise program shown by studies to reduce injury rates. Such a system could also be used to collect data to make further study more effective.

Efforts should also be explored to further study and implement findings about the potential protective effects of stretching regimens and plyometric and strengthening exercises because some studies have shown significant reductions in injury risk after implementing programs, but optimized and standardized approaches have not yet been established sufficiently. One potential area that shows promise is using machine-learning to predict weaknesses and risks that could be addressed on a player-specific basis [12]. Sharing evidence with young athletes that participating in plyometric training can improve their kicking distance, agility, jumping ability, and sprint times in addition to preventing injury [5] may increase motivation and buy in.

Finally, evidence supports that higher ratios of “free play” to organized sport protect young athletes, suggesting that athletic activity itself is not what predisposes young soccer players to injury risk, but rather the specifically repetitive and regimented play involved in “organized sport” [8,15]. It also suggests that protecting and incentivizing time for free play could be a cost-effective and near universally applicable way of preventing injuries in young athletes that both public health authorities and sports governance bodies should be involved in [8]. The implications of sport specialization versus participation in multiple sports warrant further investigation, as numerous youth athletes engage in various sports, while elite athletes tend to exhibit greater specialization. These differing activity patterns may result in distinct injury risk profiles among these groups.

There are several limitations of this article including that some studies focused on elite athletes could lack generalizability to others in the target population. The population of youth soccer players is also diverse and difficult to study in a controlled manner that would allow for definitive establishment of causation in injury risk analysis. There is also no centralized organization that could collect data for research or implement suggestions made by researchers. Given these limitations, factors that have been established to correlate with injury risk across multiple studies and not contradicted by others may be the most concrete type of data plausible and can be considered a reasonable basis for suggesting interventions.

## 5. Conclusions

Adolescent soccer players are at risk for overuse injuries, particularly during periods of rapid growth including PHV. There are multiple nonmodifiable anatomic risk factors that allow assessment of periods of increased risk, and many modifiable risk factors such as training type, volume, intensity, sport specialization that can be mitigated by appropriately targeted prevention strategies. Evidence supports neuromuscular training and structured monitoring as promising preventive strategies, but there remains a lack of standardized, evidence-based protocols. Future research should focus on optimizing and validating interventions, integrating growth and load monitoring, and leveraging emerging approaches such as machine learning-based risk prediction.

We encourage parents, coaches, youth sports organizations, and the health care community to implement evidence-based interventions to decrease the burden of injury on adolescent athletes and support their healthy development and soccer performance.

## Figures and Tables

**Table 1:** Studies Included and Major Findings.

Source	Outcome Measures	Summary and Major Findings
Dupré et al., 2020 [7]	Causes/Risks	Kinematic data was captured for 60 athletes performing short passes. Hip adductor muscle forces increased with age with the largest increase (49%) between U12 and U15. Strength increased with increased lower limb mass.
Frome et al., 2019 [8]	Causes/Risks	Retrospective survey of 2099 elite youth male soccer players (average age 13.2 ± 1.8 years), 61.7% played soccer >8 mo/yr and no other sports (specialized) and 38.3% played soccer <8 mo/yr and also played other organized sports. Both groups had similar rates of overuse injuries. Specialized athletes missed more practice due to injuries. Players who spend more than twice as much time on organized sport vs. free play were at 35% increased risk of injury.
Johnson et al., 2020 [4]	Causes/Risks	76 talented young football players were analyzed prospectively over two competitive seasons. The period around peak height velocity (PHV) (24.5 injuries per 1000 h) was associated with a significantly higher injury incidence rate and burden compared to pre-PHV (11.5 injuries per 1000 h). No difference in injury risk comparing early- vs. late-maturing athletes.
Leppänen et al., 2019 [9]	Causes/Risks	733 players from 10 different clubs in Finland were followed prospectively. 46.8% of players reported an overuse problem. The average weekly prevalence of all overuse problems and substantial overuse problems was 12.8% and 6.0%, respectively. Injuries affecting the knee had the highest weekly prevalence (5.7% for all and 2.4% for substantial knee problems). Girls had a higher likelihood of knee problems (OR 2.70; 95% CI 1.69 to 4.17) while boys had a higher likelihood of heel problems (OR 2.82; 95% CI 1.07 to 7.44).
Leppänen et al., 2022 [10]	Causes/Risks	447 male and female players aged 9 to 14 years (median 12 years) participated in performance tests and prospective follow-up. A high level of composite physical fitness was associated with an increased rate of all injuries RR = 1.28.
Mandorino et al., 2023 [11]	Causes/Risks	A systematic review of studies focused on injury epidemiological data and risk factors in youth soccer players. Several studies found a higher injury incidence during the second half of the match, especially the last 15 min. High internal training loads were linked to increased risk of injuries, and lower preseason aerobic fitness training was linked to higher risk of injury during the season. Two studies found early maturing players to be at higher risk of injury using PHV measurements.
O’Kane et al., 2017 [12]	Causes/Risks	3351 female youth soccer players, ages 12 to 15 years, were evaluated prospectively from 2008 to 2012. Incidence rate for first-time lower extremity overuse injuries was 1.7 per 1000 athlete-exposure hours (AEH). The incidence of repeat injuries was 3.4 per 1000 AEH. A 1–standard deviation (SD) increase in hamstring strength was associated with a 35% decreased risk of overuse of knee injuries. A 1-SD increase in quadriceps strength was associated with a 30% decreased risk of overuse injuries. A 1-SD increase in hip flexor strength was associated with a 28% decrease in risk of overuse injuries. 1-SD increase in external rotation strength was associated with a 35% decreased risk of overuse injury. Playing on more than 1 soccer team was associated with a 2.5-fold increased risk (95% CI, 1.08–5.35) for overuse knee injuries, and participating in other physical activities was associated with a 61% decreased risk.
Petry et al., 2016 [13]	Causes/Risks	Observational study of 118 players found decreased postural stability after completing a soccer training session.
Read et al., 2016 [2]	Causes/Risks	Literature review of injury patterns among male youth soccer players, highlighting the predominance of lower extremity injuries, particularly ligament sprains at the ankle and knee. The authors emphasize the need for further research into injury mechanisms and propose a hierarchical model for assessing and addressing injury risks in male youth soccer players.
Read et al., 2016 [14]	Causes/Risks	This literature review found that early sport specialization has shown only limited benefits in developing elite soccer players and can lead to skeletal deformities due to disrupted growth. The study recommended the entry age into formalized academy soccer programs may be most beneficial after age 12 years, and deliberate play should be emphasized prior to this point.
Read et al., 2018 [15]	Causes/Risks	608 elite male youth soccer players aged 11–18 years from the academies of six professional soccer clubs were prospectively monitored, recording injuries during the 2014–2015 soccer season. U14 and U15 age groups were most prone to injury; ankles and knees were the most common joints injured, and injuries peaked at the start of the season in September and in January.
Robles-Palazón et al., 2022 [16]	Causes/Risks	314 elite youth (10–19 years) soccer players were followed prospectively for a 9-month competitive season. The hamstring was commonly injured, hamstring injuries were particularly burdensome, increased age and maturity increased injury risk, and PHV was associated with increased overuse injury risk.
Rommers et al., 2020 [6]	Causes/Risks	Prospective cohort study of 314 elite players (11.7 ± 1.7 years of age). 160 players sustained 133 overuse and 163 acute injuries. In the U10–U12 group, risk of overuse injuries was associated with an increase in leg length over the season (incidence rate ratio (IRR) 1.620 [95% CI 1.230–2.117]). In the U13–U15 group, a higher leg length was associated with an increased risk of overuse injuries (IRR 1.055 [95% CI 1.011–1.108]).
van der Sluis et al., 2015 [17]	Causes/Risks	26 soccer players (mean age 11.9 ± 0.84 years) were followed prospectively for 3 years. Peak Heigh Velocity (PHV) was calculated using the Maturity Offset Protocol. Later maturing players had a significantly higher incidence of overuse injuries than earlier maturing players both in the year before PHV (3.53 injuries/1000 h of exposure vs. 0.49 injuries/1000 h in the control group; *p* < 0.05) and during the year of PHV (3.97 injuries/1000 h of exposure vs. 1.56/1000 h in the control; *p* < 0.05)
van der Sluis et al., 2014 [18]	Causes/Risks	26 male soccer players (mean age: 11.9 years) were observed prospectively over a 3-year period around their PHV. Overuse injuries increased during and after PHV. Players missed significantly more days due to injuries during PHV (15.69 days per year) compared to the year before PHV (7.27 days).
Wada et al., 2024 [19]	Causes/Risks	35 youth soccer players aged 12–15 years were studied retrospectively. Inertial measurements of the player’s thoracic and lumbar spine, pelvis, thigh, and lower leg as well as sagittal plane tilt of each body segment while squatting were recorded. Osgood-Schlatter Disease correlated with lower lumbar and pelvic coordinated variability, and the authors theorized that increased load on the knee joint extension mechanisms may be part of the mechanism of injury.
Zoellner et al., 2022 [20]	Causes/Risks	414 youth football players aged 10–15 years were surveyed retrospectively in New Zealand. Highly specialized participants were 4× more likely to report “gradual onset injuries” and injuries overall than those who were low specialized. Gradual onset injury was also associated with playing football for more than 8 months of the year and increased weekly organized sport participation volume, regardless of level of specialization.
Bedoya et al., 2015 [5]	Prevention	Plyometric exercises enhance athletic performance by utilizing the stretch-shortening cycle (SSC), which improves power by engaging both the elastic properties of muscles/tendons and the stretch reflex. Training programs of 8–10 weeks, conducted twice per week with 72 h rest intervals, were most effective. Programs should start with 50–60 foot contacts per session, progressing to 80–120 contacts, with 2–4 sets of 3–4 plyometric exercises per session, following recommended safety guidelines.
Cheng et al., 2019 [21]	Prevention	Prospective cohort study of 177 female soccer players age 10–18. BMI and reaching menarche were significant injury risks but screening hip exams did not predict any injuries.
Imai et al., 2018 [22]	Prevention	Two teams were observed prospectively: one performing trunk stabilization exercises (INT) before practice and games, while the control team (CON) followed their usual warm-up. Overall injury rates were significantly lower in the INT team (2.65 injuries/1000 h) compared to the CON team (4.94 injuries/1000 h), with an incidence rate ratio (IRR) of 0.54.
Rommers et al., 2020 [23]	Prevention	A machine learning model proved effective in predicting injuries and classifying them as overuse or acute based on preseason physical attributes. Findings differ from traditional statistical models due to the ability to incorporate a larger number of variables. The study identified that higher body height, leg length, and predicted age at PHV were significant risk factors for injuries. Motor performance factors like flexibility (sit-and-reach test) had a small protective effect, contrasting previous studies in adults. The models allow for injury risk management strategies tailored to individual players, potentially improving resource allocation in injury prevention.
Salter et al., 2021 [24]	Prevention	Forty-nine support staff from male Premier League academies and female Regional Talent Clubs completed a survey on monitoring of biological maturity and training load and injury prevention. Results indicate discrepancies in methods for estimating biological maturity and tracking training loads. Although injury prevention is a top priority, barriers such as resource limitations, staff numbers, and communication issues affect the consistency and effectiveness of monitoring practices. The study highlights the need for more standardized protocols and better stakeholder communication to mitigate injury risks.
Tears et al., 2018 [25]	Prevention	Prospective study of a new development strategy called Elite Player Performance Plan for preventing injury incidence and patterns in elite youth soccer players. Exposed athletes to more coaching hours and growing players strength on home turf. The study found a lower injury burden in the U12–U15 group after adopting the program contrasted with an increased injury burden in the U16–U18 group.
Wollin et al., 2018 [26]	Prevention	22 male players age 15.53 ± 0.48 years were investigated prospectively. Adductor squeeze strength was measured daily throughout a 7-game international tournament. Each male player had a past groin injury. 45% of players reported groin pain and athletes who measured higher adductor peak force were less likely to report groin pain.
Babler et al., 2025 [3]	Location/Type	Review of the types of injuries in female athletes and their prevalence. Overuse injuries are less prevalent than traumatic injuries, accounting for less than 25% of all female soccer injuries. Overuse injuries include apophysitis, patellofemoral pain syndrome, bone stress injury, sesamoiditis, and iliotibial band syndrome.
Belikan et al., 2022 [27]	Location/Type	Retrospective evaluation of 4326 total injury cases in 612 players over 10 years. 0.36 cases of calcaneal apophysitis per 100 athletes per year were observed in players with a mean age of 11.8 ± 2.1 years and resulted in mean time to return-to-play of 60.7 ± 64.9 days. Recurrent pain was associated with longer recovery time and time to return to play.
Suzue et al., 2014 [28]	Location/Type	1162 players at a soccer tournament were surveyed retrospectively, with 47.1% (547 players) reporting pain, primarily in the lumbar spine or lower extremities. Apophysitis was prevalent in the examined players, with most diagnoses being related to overuse injuries, particularly in the heel and knee.

## Data Availability

No new data were created or analyzed in this study.

## Appendix A. Literature Search Strategy and Selection Criteria

A comprehensive search was performed in PubMed and Embase to identify studies related to overuse injuries among adolescent soccer players.

PubMed Search String: (“Cumulative Trauma Disorders”[Mesh] OR “cumulative trauma disorder*” OR “repetitive stress injur*” OR “repetition strain injur*” OR “repetitive motion disorder*” OR “repetitive strain injur*” OR “overuse syndrome” OR “overuse injur*”) AND (“Soccer”[Mesh] OR soccer OR football*) AND (“Adolescent”[Mesh] OR adolescen* OR youth OR young OR teen* OR school* OR “Pediatrics”[Mesh] OR pediatric* OR paediatric*)

Embase Search String: ‘cumulative trauma disorder’ AND (soccer OR football) AND (adolescent OR juvenile OR pediatric) AND [embase]/lim NOT ([embase]/lim AND [medline]/lim)

The search was conducted from March 2014 to 2024, without language restrictions. Gray literature, conference abstracts, and non-peer-reviewed reports were excluded to ensure methodological rigor.

Inclusion Criteria

- Studies evaluating overuse injuries (including tendinopathy, apophysitis, stress reactions, and chronic musculoskeletal complaints) in adolescent soccer players (ages 10–19).
- Original research studies, including cohort, case–control, and cross-sectional designs.
- Studies reporting epidemiological outcomes, risk factors, clinical presentation, or management strategies.

Exclusion Criteria

- Studies focusing exclusively on acute injuries (e.g., fractures, ligament tears, concussions).
- Non-soccer athletic populations or mixed cohorts where soccer-specific data could not be isolated.
- Case reports, editorials, commentaries, and conference abstracts.
- Studies without sufficient methodological detail to evaluate outcomes.
